# Impact of cardiometabolic risk factors and its management on the reversion and progression of arterial stiffness

**DOI:** 10.1038/s44325-025-00074-6

**Published:** 2025-07-08

**Authors:** Natalia Sofía De la Maza-Bustindui, Mariana León-Álvarez, Camila Ponce-Acosta, Kathya Paola Zarco-Morales, Carlos Alberto Fermín-Martínez, Neftali Eduardo Antonio-Villa, Omar Yaxmehen Bello-Chavolla

**Affiliations:** 1 Research Division, Instituto Nacional de Geriatría, Ciudad de México, Mexico; 2 Facultad de Medicina, Universidad Nacional Autónoma de México, Ciudad de México, Mexico; 3 Facultad de Medicina, Universidad La Salle, Ciudad de México, Mexico; 4 MD/PhD (PECEM) Program, Facultad de Medicina, Universidad Nacional Autónoma de México, Ciudad de México, Mexico; 5 Departamento de Endocrinología, Instituto Nacional de Cardiología Ignacio Chávez, Ciudad de México, Mexico; 6 Department of Global Health and Population, Harvard T.H. Chan School of Public Health, Boston, MA USA

**Keywords:** Cardiology, Endocrinology

## Abstract

Arterial stiffness is associated with a higher risk of adverse cardiovascular outcomes. Cardio-metabolic diseases increase the risk and progression of arterial stiffness, and its optimal management along with lifestyle interventions may decrease its impact on the risk of cardiovascular outcomes. In this review, we highlight recent evidence on the impact of cardiometabolic risk factors and their management on arterial stiffness and identify potential areas of opportunity for future research.

## Introduction

Cardiovascular disease (CVD) is the leading cause of death worldwide^1^. Among the many factors contributing to the development and progression of CVD, arterial stiffness (AS) has gained significant attention in recent years due to its strong association with a range of relevant CVD outcomes, including stroke, myocardial infarction, and heart failure^2,3^. AS is a crucial marker in cardiovascular health, as it reflects the functional state of arteries^4^, and previous research has shown it may be a useful predictor for increased risk of adverse cardiovascular outcomes in clinical practice^5^. Therefore, AS can be conceptualized as a cardiovascular risk factor which can be assessed non-invasively in clinical practice, and which can also serve as a marker to monitor the effect of cardiovascular health interventions^4–6^.

Inflammation and vascular aging are key factors involved in large artery stiffening, contributing to atherosclerosis and arteriosclerosis^3^. AS is often the result of multiple pathophysiological processes which are commonly involved in the development and progression of CVD, including endothelial dysfunction, vascular smooth muscle cell (VSMC) migration and proliferation, vascular calcification, extracellular matrix (ECM) remodeling, oxidative stress and proinflammatory processes^7^. Furthermore, many of these are also related to the pathophysiology of cardiometabolic diseases, including insulin resistance (IR), adipose tissue dysfunction, dyslipidemia, impaired kidney function, and type 2 diabetes mellitus (T2D), which are all encompassed within the cardiovascular-kidney metabolic syndrome (CKMS)^4,6^; therefore, it is pivotal to assess the metabolic and aging-related contributions of cardio-metabolic conditions to vascular health^5^. Moreover, whilst the association between cardiometabolic disease and AS is well-established^8^, the effect of mitigating cardiometabolic risk factors on AS remains an active area of research. In this narrative review, we explore the pathophysiological links between AS and cardiometabolic diseases, examining evidence on whether managing these diseases and their risk factors influences AS development and progression. Additionally, we highlight potential research opportunities and address unresolved questions in the current literature.

## Definition and measurement of arterial stiffness

### Definition and relevance of arterial stiffness

AS stems from degeneration of the arterial wall, as a result of arterial thickening and calcification, accompanied by a gradual loss of elasticity^9^. These changes within AS can be conceptualized as hallmarks of vascular aging; moreover, they can also be related to pathophysiological changes from chronic cardio-metabolic diseases^7,9^. Healthy, elastic arteries expand to accommodate blood ejected during systolic contraction, generating pulse waves that propagate through the arterial circulation. The velocity of these waves indicates arterial elasticity, with higher velocity reflecting stiffer vessels^9^. AS increases pulse wave velocity (PWV) during systole, which, combined with reduced reserve function, can elevate blood pressure (BP), particularly systolic BP and pulse pressure. Over time, arterial wall thickening increases stiffness and PWV during systole, consequently impairing vascular hemodynamics^4,9^. AS also impairs the ability of arteries to adapt to pressure changes during the cardiac cycle^4,9,10^. Large arteries, such as the aorta, play a crucial role in buffering BP and blood flow fluctuations, protecting the microvasculature of target organs^10^.

Aging, coupled with environmental, genetic, and social factors, promotes AS, which can lead to isolated systolic hypertension, left ventricular (LV) remodeling, and cardiac dysfunction^4,9^. These changes have been demonstrated in longitudinal studies were it has been shown that the stiffness of the large arteries leads to a significant increase in BP and systemic arterial hypertension (SAH)^11–13^; similarly, evidence from the Framingham Heart Study suggests that AS is an independent risk factor for the onset of multiple long-term adverse outcomes, including SAH, T2D, chronic kidney disease (CKD), dementia, CVD, and all-cause mortality^14^. AS also negatively affects cerebrovascular function, contributing to decreased cerebral blood flow and endothelial dysfunction, potentially increasing the risk of stroke^15^. Notably, the use of medications for common cardiometabolic diseases results in beneficial effects on AS^4^. Given its well-established relationship with cardiometabolic risk, there is growing interest in using AS as a non-invasive marker of cardiometabolic disease progression and treatment benefit; however, the integration of AS measures into routine clinical practice is not yet common, as discussed in length in later sections^16^.

### Non-invasive measurement of arterial stiffness

AS can be measured at systemic, local, and regional levels. Systemic measurements of AS can only be obtained using circulation models; however, regional and local data can be assessed through non-invasive procedures^17^. The most commonly used method to measure aortic AS is the determination of PWV using either Magnetic Resonance Imaging (MRI) or tonometry of the right carotid and femoral arteries using the “foot-to-foot” velocity method (carotid-femoral PWV; cf-PWV), or the brachial-ankle PWV (ba-PWV), often with electrocardiogram as a timing marker^17–19^. PWV represents the velocity at which the BP pulse wave travels through the arteries, and it can be calculated using the Bramwell-Hill equation, which defines the relationship between arterial distensibility and PWV^20^. In clinical practice, PWV is often determined as the ratio of the distance between two measurement sites to the time it takes for the pulse to travel between them^17,19^. In addition to cf-PWV, ba-PWV, and MRI-based PWV, other non-invasive methods to assess AS include the cardio-ankle vascular index (CAVI), the augmentation index (AIx), and the measurement of carotid intima-media thickness. These methods are briefly described along with their potential advantages and disadvantages in Table 1.

**Table 1:** Non-invasive methods for the assessment of arterial stiffness in research and clinical practice

Measurement method	Region of measurement	Description	Advantages and disadvantages	Source
Carotid-Femoral Pulse Wave Velocity (cf-PWV)	Carotid and femoral arteries	The most widely used method. It measures the speed at which the pulse wave propagates between the carotid and femoral arteries.	Highly reliable for central arterial stiffness; however, it does not assess the ascending aorta.	Castelli et al. ^2^
Brachial-Ankle PWV (ba-PWV)	Brachial and ankle arteries	It measures PWV from the brachial artery to the ankle.	Useful for screening in larger populations, but it has important variability due to arterial heterogeneity.	Dupont et al. ^19^
MRI-based PWV	Aortic segments	Allows for more precise measurements in specific segments of the aorta that cannot be assessed with cf-PWV.	It has high anatomical accuracy; however this method is expensive, time-consuming and not always available.	Forcada et al. ^22^
Cardio-Ankle Vascular Index (CAVI)	Brachial and ankle arteries	Evaluates arterial stiffness independently of blood pressure, analyzing both elastic and muscular arteries. It is a predictor of incident CVD but is limited in conditions like aortic stenosis and atrial fibrillation.	It has high reproducibility and can be measured independently of BP, but it is limited in cases like atrial fibrillation or peripheral artery disease.	Budoff et al. ^18^
Augmentation Index (AIx)	Radial or carotid artery	Uses pulse wave reflection in the radial or carotid arteries to determine stiffness, considering factors such as systolic BP and the cardiac cycle.	Rapid and easy method, requires additional measures such as BP	Oliveira: et al. ^17^
Carotid Intima-Media Thickness	Carotid artery	A subclinical atherosclerosis marker that measures the thickness of the intima-media layer in the carotid artery that predicts CVD events.	It helps predict subclinical atherosclerosis; however, it is less specific for central arterial stiffness.	Mozos et al. ^3^

*PWV* Pulse wave velocity, *MRI* Magnetic resonance imaging, *cf-PWV* Carotid-Femoral Pulse Wave Velocity, *ba-PWV* Brachial-Ankle PWV, *CVD* Cardiovascular disease, *AIx* Augmentation Index,*BP* blood pressure.

## Pathophysiological mechanisms and determinants of arterial stiffness

### Pathophysiology of arterial stiffness

**Fig. 1:**
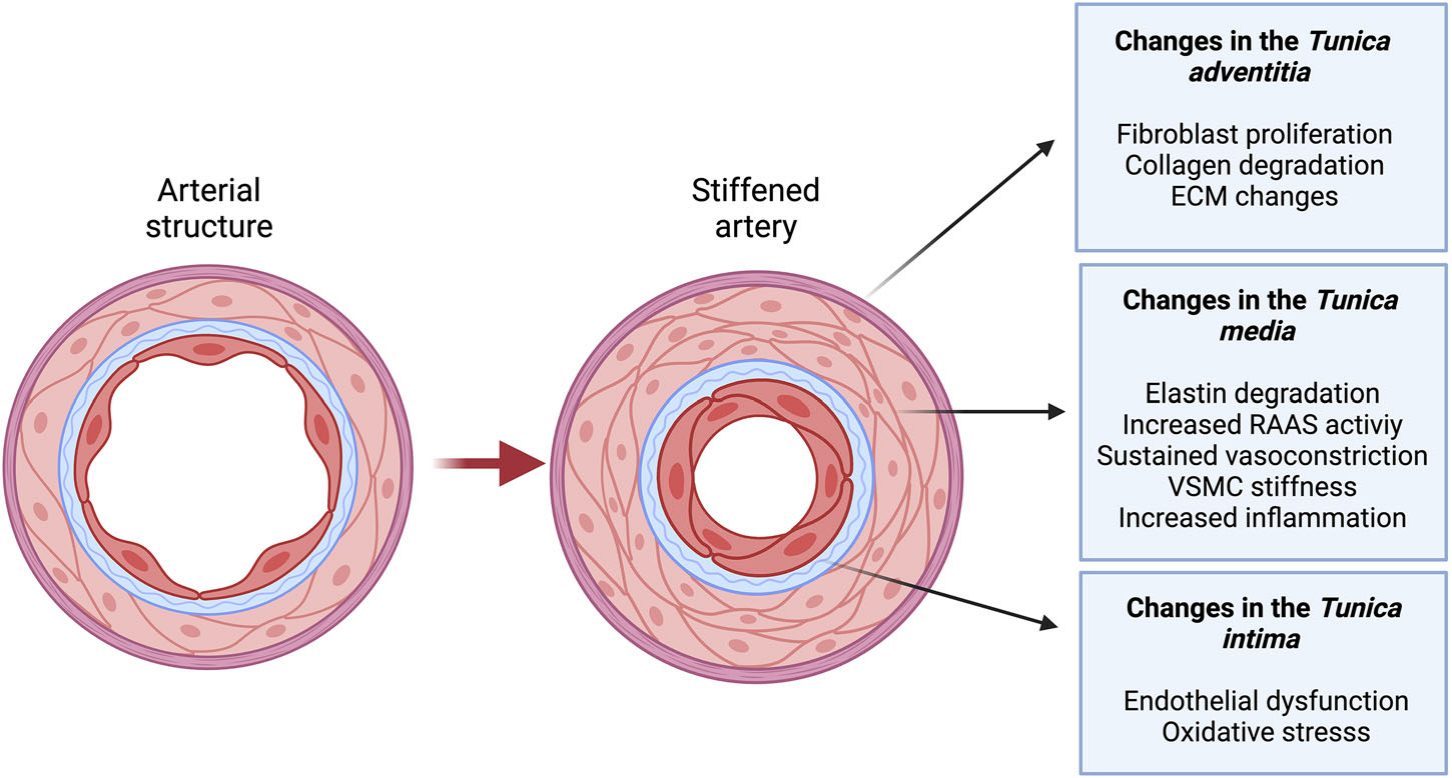
Schematic representation of the pathophysiologic changes observed in AS, highlighting the histological transitions observed within each layer of the arterial wall as AS progresses. Changes observed in each layer can occur regardless of the underlying etiology of arterial stiffness. Created in BioRender. Bello Chavolla, O. (2025) https://BioRender.com/o88u142. ECM extracellular matrix RAAS renin-angiotensin-aldosterone system VSMC vascular smooth cells AS arterial stiffness.

Mechanisms underlying AS include changes in contractility and plasticity of VSMC, arterial wall thickening, degenerative changes in arterial wall elastic fibers, ECM changes, and increased immuno-inflammatory stimuli, all of which induce distinct impacts on specific arterial layers^9^. Elastic arteries are composed of three layers: 1) the outer layer, also known as *tunica adventitia*, mainly composed of fibroblasts, collagen-containing matrix tissue, and the external elastic lamina, 2) the middle layer, also known as *tunica media*, mainly composed of VSMC and elastic fibers, and lastly, 3) the inner layer, also known as *tunica intima*, mainly composed of a single layer of endothelial cells with a basement membrane or internal elastic lamina^19^. During vascular aging, collagen deposition in the *tunica adventitia* and *media* increases as fibroblast activity rises and elastin degradation accelerates^2,7,16^. The activation of the renin-angiotensin-aldosterone system (RAAS), overstimulation of angiotensin-II type 1 and mineralocorticoid receptors, and increased expression of α-smooth muscle actin and β1-integrin contribute to VSMC stiffness^7,9^. Meanwhile, persistent oxidative stress leads to important and irreversible endothelial dysfunction in the *tunica intima*. Figure 1 summarizes how these processes collectively contribute to the onset of AS. The pathophysiology of AS is conditioned by structural, dynamic, and external components^4,21^. Structural factors include impairments in collagen and elastin fibers in the *intima media*, as well as blood vessel calcification. Dynamic factors are characterized by increased stimulation of VSMCs by vasoactive substances, while external components are primarily modifiable risk factors^22^. The events that contribute to the development of AS are outlined in Table 2.

**Table 2:** Summary of structural, dynamic and external pathophysiological mechanisms characterized in arterial stiffness

Structural component: Arterial wall remodeling	Impaired collagen-elastin ratio	Type I collagen fibers increase and the number of elastic fibers and VSMC decrease, primarily by overstimulation of elastase and reduction of tropoelastin expression.
	Blood vessel calcification	Inflammation, oxidative stress, and the presence of cardiometabolic comorbidities decrease mitophagy and autophagy, and stimulate vascular calcification, which decreases vascular distensibility.
	Endothelial dysfunction	Inflammation can cause disruptions in the regulation of vascular tone, leading to vascular dysfunction and irreversible vessel damage.
	Increased intima-media thickness	Caused by VSMC migration, endothelial dysfunction, vascular calcification, increased activity of metalloproteinases, ECM degradation, oxidative stress, collagen and elastin degradation.
Dynamic component: Stress	Proinflammatory markers	Inflammation promotes activation of RAAS, the sympathetic nervous system and expression of endothelin-1, which leads to overproduction and increased secretion of MCP-1, TGF-β, matrix metalloproteinases, calpain-1, and MFG-E8, leading to age-associated arterial secretory phenotypes.
	Reactive oxygen species	Caused by increases in the production of ROS and in the expression of NADPH in the arterial wall, as well as a negative regulation of antioxidant proteins during vascular aging, such as superoxide dismutase.
	Nitric Oxide	NO works as a regulator for vasodilation, stiffening and proinflammation. Decreases in NO synthase and NO concentrations in the arterial wall induce a proinflammatory reaction, generating ROS and increasing vasoconstriction.
External components		• Clinical risk factors (T2D, systemic arterial hypertension, obesity). • Genetic determinants. • Behavioral risk factors (smoking, alcoholism).

*VSMC* Vascular smooth muscle cells, *CVD* Cardiovascular disease, *ECM* extracellular matrix,*RAAS* renin-angiotensin-aldosterone system, *ROS* reactive oxygen species, *NADPH* nicotinamide adenine dinucleotide phosphate (reduced), *NO* Nitric Oxide, *MCP-1* Monocyte Chemoattractant Protein-1, *TGF-β* Transforming Growth Factor Beta, *MFG-E8* Milk Fat Globule-Epidermal Growth Factor 8, *T2D* Type 2 Diabetes.

### Ethnic and genetic variation in arterial stiffness and its impact on CVD risk

AS exhibits significant heritability, likely influenced by a combination of genetic and environmental factors^4,9,10^. Ethnic and socioeconomic factors also play significant roles in AS. For instance, AS has been shown to be more prevalent and progresses earlier in African and Hispanic compared to Caucasian-descent populations, leading to early vascular aging, and consequently, an increased propensity for AS^23^. While genetic predisposition contributes to AS, lifestyle factors, socioeconomic adversity, and psychosocial stress further modulate these risks and its prognostic value for AS^24–26^. Socioeconomic status and health behaviors, such as obesity, are also contributors to AS, particularly in communities facing economic adversity^23,25^. Conversely, metabolomic studies have identified urinary amino acids involved in collagen metabolism and oxidative stress regulation which are protective of AS in African American children against early vascular deterioration^27,28^. These factors underscore the necessity of addressing social and biological determinants of health to better understand AS as both a clinical entity, and as a CVD risk factor^24^.

Recent studies have provided insights into the molecular underpinnings of AS, particularly through transcriptional profiling, highlighting the involvement of genes associated with ECM remodeling and VSMC function^10^. For example, aortic biopsies from patients with AS reveal alterations in genes regulating vascular structure, signaling, and gene expression, including downregulation of *PPP1CB* and *PRKA*, which are critical for VSMC contraction, and upregulation of the gene encoding phosphoinositol-3-kinase polypeptide p85α^29^. Overall, three predominant gene expression patterns have been associated to AS, including 1) genes that promote contractile and relaxant properties as well as intercellular VSMC signaling, 2) genes and inhibitors which regulate VSMC growth, and 3) genes encoding structural proteins like elastin and type III collagen that directly impact AS via ECM remodeling^29–31^. The integration of *omics* approaches, including genomics, metabolomics, and microbiota composition, offers a promising avenue for identifying molecular pathways and mechanisms of AS in humans^9,29^. Integration of these approaches could facilitate the development of targeted therapies to mitigate the associated cardiovascular risk related to AS and help characterize the complex interplay of biological, social, and psychosocial factors involved in its pathophysiology^32,33^.

## Arterial stiffness in the pathophysiology of cardiometabolic diseases

AS plays a significant role in the pathophysiology of metabolic diseases, particularly within the framework of the cardiovascular-kidney metabolic syndrome (CKMS), as highlighted by the American Heart Association’s Disease Continuum framework^34^. CKMS encompasses a variety of related cardiometabolic risk factors, including obesity, elevated BP, IR, abnormal blood lipid levels, and impaired kidney function^6,34,35^ in patients with or without over CVD. The spectrum of CKMS includes the most relevant cardiometabolic diseases and risk factors and is a useful framework to guide management and policy^36^. CKMS considers the transition from patients without cardiometabolic risk factors (stage 0), to those with excess or dysfunctional adiposity (stage 1), those with cardiometabolic risk factors including hypertriglyceridemia, SHA, T2D, metabolic syndrome, or CKD (stage 2), those with subclinical CVD, high or very high risk of CVD with additional cardiovascular, kidney or metabolic risk factors (stage 3), and those with clinical CVD in CKMS (stage 4)^37^. AS arises in CKMS as a result of pathophysiological changes which lead to an imbalance in the elastin-to-collagen ratio, inflammation from ROS, vascular calcification, VSMC stiffening, and endothelial dysfunction resulting from underlying metabolic and inflammatory processes^6,34^. Notably, AS can occur across CKMS stages 1–4, starting from states of excess of dysfunctional adiposity and in patients with over clinical CVD. Moreover, AS favors the development of CVD through several mechanisms. Pulse waves travel faster in stiffened arteries, combining with the reflected wave in the early stages to increase systolic and decrease diastolic BP, thus increasing pulse pressure^18^. These BP changes may lead to LV hypertrophy due to increased myocardial strain, diastolic dysfunction and a reduction in coronary artery blood flow, favoring ischemia^10,38,39^. Furthermore, increased mechanical shear due to increased pulse pressure facilitates lipid accumulation in the arterial wall and weakening of the fibrous cap in atherosclerotic lesions^40^.

**Fig. 2:**
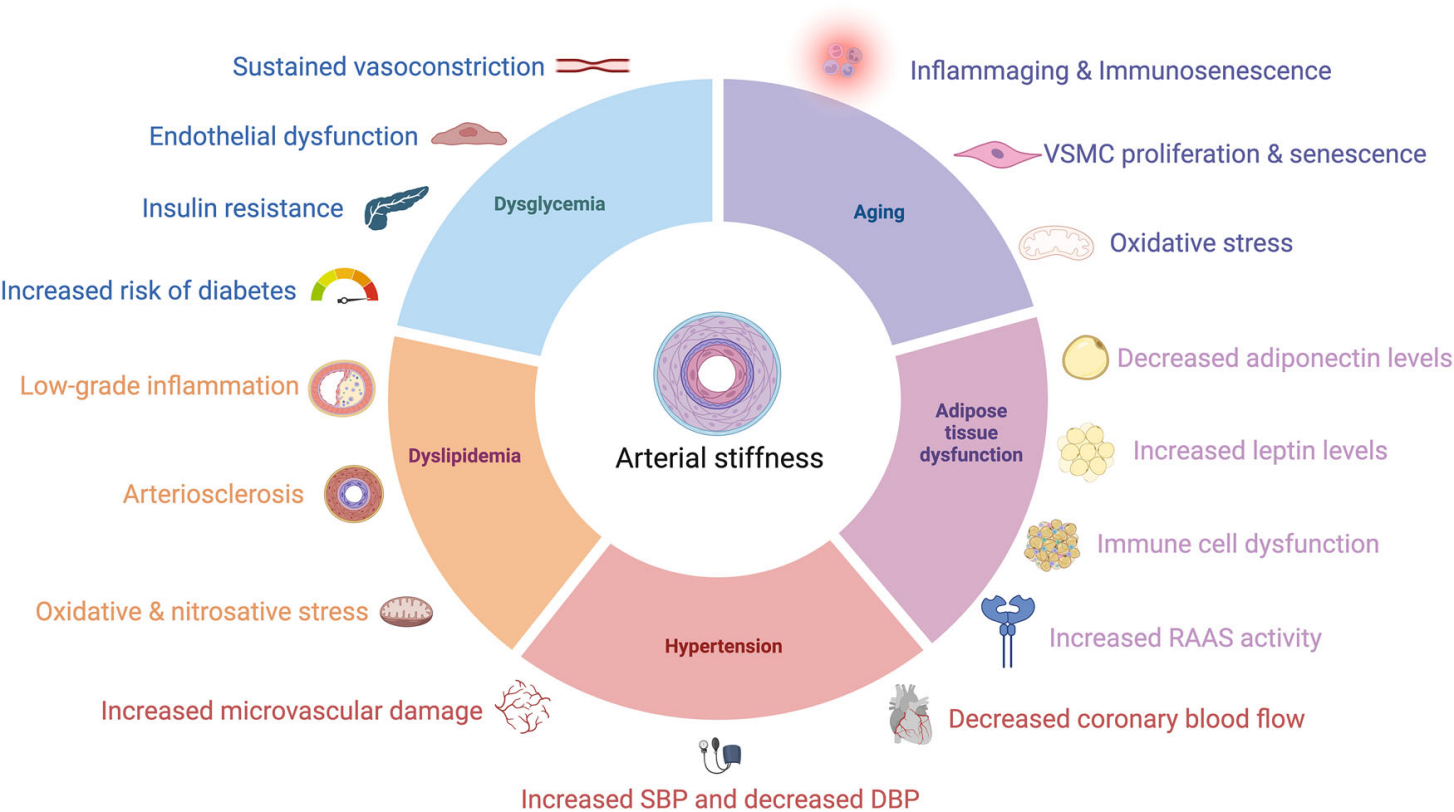
Mechanisms involved in the pathophysiology of cardio-metabolic diseases which contribute to the onset and progression of AS. The figure highlights five main etiologic domains which are extensively discussed in the text, including aging, dysglycemia, adipose tissue dysfunction in obesity, dyslipidemia and arterial hypertension. The main mechanisms involved in each etiologic domain are highlighted with the same color. Created in BioRender. Bello Chavolla, O. (2025) https://BioRender.com/p44w501. RAAS renin-angiotensin-aldosterone system VSMC vascular smooth cells AS arterial stiffness SBP systolic blood pressure DBP diastolic blood pressure.

The relationship between cardiometabolic risk factors and long-term patterns in the progression of AS can vary significantly^41^. Risk factors contributing to elevated PWV include male gender, smoking, and metabolic disruptions in glucose and lipid levels^9^. Additionally, prolonged exposure to tobacco, excessive alcohol intake, SAH, T2D, hypertriglyceridemia, and hyperuricemia are independently associated with a rapid rise in AS, especially in older individuals^41,42^. Although AS tends to increase with age, this progression is faster in people with obesity, IR, and diabetes^6,34,43,44^; furthermore, aging increases the risk of CKMS and its associated comorbidities, further contributing to AS progression^43,45^, thus increasing the risk of CVD, kidney dysfunction, and stroke. Previous studies showed that an increase of 1 m/s in estimated PWV was associated with ~30% higher risk of CVD outcomes and death^46^. Currently, the protective factors against this damage remain unclear^4^. Figure 2 resumes the main pathophysiological mechanisms found in metabolic diseases which increase the risk of AS onset or progression, and which will be described in the following sections. Data from prospective and longitudinal studies have demonstrated the long-term impact of PWV and AS on relevant cardiometabolic markers and outcomes. Notably, AS has been associated with an increased risk of new-onset T2D, SAH, CVD, stroke and dementia, and increased PWV has been consistently associated with worsened management of cardiometabolic risk factors and increases risk of long-term complications for patients living with cardiometabolic diseases. A detailed summary of prospective evidence regarding the relevance of AS and PWV for cardiometabolic diseases from relevant and large-scale studies is summarized in Table 3.

**Table 3:** Summary of evidence from relevant prospective and longitudinal studies outlining the relevance of AS and PWV for patients living with cardiometabolic diseases and risk factors

Study	Outcomes	Length of follow-up	Evidence	Ref.
Metabolic and cardiovascular disease				
UK Biobank	Diabetes	9.5 years	AS increases the risk of diabetes in a dose-response fashion, especially in participants at high genetic risk of diabetes.	^170^
Framingham Heart Study / UK Biobank	Diabetes	7 years	AS increases risk of T2D independent of BP	^171^
Framingham Heart Study	Hypertension, CKD, diabetes, dementia, CVD, stroke, and AF.	15 years and 7.1 years for AF	AS increases risk of hypertension, CKD, diabetes, dementia, CVD, stroke and atrial fibrillation. Increased PWV may precede the onset of AF.	^14,172^
Framingham Heart Study	Hypertension and CVD	13 years	~32% increase in CVD per 1-SD increase in PWV. SAH and AS jointly increased risk of CVD.	^173,174^
Kailuan Cohort	Diabetes	3.72 years / 6.16 years for SAH + AS	AS increases risk of T2D and precedes increases in FPG. Participants with SAH and AS and normotensive with AS had the highest risk of diabetes.	^61,87^
TRIPLE-A-Stiffness	Cardiovascular and all-cause mortality	3.82 years	CAVI was associated with increased risk of cardiovascular and all-cause mortality, independent of additional risk factors.	^175^
Multi-Ethnic Study of Atherosclerosis (MESA)	Cardiovascular disease	16 years	PWV and AS increases risk of myocardial infarction, resuscitated cardiac arrest, stroke, adjudicated angina, and cardiovascular death	^176^
SardiNIA study	Multiple organ damage	9.0 years	Each 1 m/s increase in PWV led to left ventricular hypertrophy, intima-media thickness >0.9 mm and/or plaque, and eGFR <60 ml/min/1.73 m2	^11^
Avon Longitudinal Study of Parents and Children	Metabolic syndrome	7 years	AS temporally preceded the onset and progression of metabolic syndrome in adolescents.	^8^
Modifying cardiometabolic factors on arterial stiffness				
Malmo Diet and Cancer Study	Arterial Stiffness	5 years	Insulin resistance, abdominal obesity, TG and HDL-C (but not LDL-C) predict PWV levels after follow-up independent of BP levels	^88,177^
Kailuan Cohort	Management of hypertension	6.0 years	Antihypertensive treatment reduces AS and precedes BP lowering.	^13^
Beijing Xiaotangshan Cohort	Diabetes and glycemic control	5 years	AS increases risk of T2D, particularly in participants with abdominal obesity. Glycemic control decreased the impact of AS on T2D	^153,178^

*AS* Arterial stiffness,*T2D* Type 2 Diabetes, *SAH* Systemic Arterial Hypertension;*BP* Blood pressure;*CVD* Cardiovascular Disease, *CKD* Chronic Kidney Disease, *AF* Atrial fibrillation, *PWV* Pulse wave velocity, *FPG* Fasting Plasma Glucose, *SD* Standard deviation, *TG* triglycerides, *CAVI*cardio-ankle vascular index.

### Arterial stiffness and aging

AS has been traditionally associated with increasing chronological age^9,44^. In early adulthood, cf-PWV increases by 0.2–0.4 m/s per decade, consecutively increasing in average by 1.2–2.4 m/s for individuals 80 years and older^47^. Notably, these PWV increases are independent of arterial BP, indicating that other factors in addition to chronological aging contribute to this late-life change^29,44^. These dynamic alterations, which can be conceptualized as hallmarks of vascular aging, are influenced by DNA damage, telomere attrition, genetic alterations, mitochondrial dysfunction, a reduced stem cell pool, and issues in protein processing^48^. These pathways result in thickening of the *intima* layer as well as low-grade inflammation (inflammaging) and decreased immune function (immunosenescence), which may also affect the *media* layer leading to AS, a higher risk of endothelial dysfunction, and subsequent risk of CVD^49^. Over time, VSMC undergoes phenotypic changes which enhance their proliferation, migration, apoptosis, and senescence^29,40,43^. Aging causes large elastic arteries, such as the aorta and carotid arteries, to enlarge and stiffen, while medium-sized distal muscular arteries tend to maintain their size and distensibility; however, hypertrophic remodeling occurs universally in these cases^44,49^. In aged arterial walls, ROS production rises significantly, primarily due to increased NADPH oxidase activity^49^. Simultaneously, antioxidant proteins in the ECM, such as copper and zinc superoxide dismutase, decrease with age^50,51^. This imbalance creates a proinflammatory environment which contributes to inflammaging and AS^3,44^. Aging, coupled with this proinflammatory state, induces structural changes in ECM structure and composition. During this process, telomere shortening and reduced telomerase activity promote DNA damage, which leads to replicative senescence; these senescent cells accumulate in various tissues, particularly affecting fibroblasts, immune system cells, and endothelial cells, contributing to decreased arterial wall elasticity and increased rigidity.

Aging is also associated with increased mental, physical, and environmental stress, as well as a decreased physiological reserve in response to external stimuli^52^. This results in increased RAAS and sympathetic nervous system activity, which enhances the release of vasoactive substances and promotes the secretion of proinflammatory cytokines and chemokines in the arterial wall leading to sustained vasoconstriction^53^. These age-related modifications have been proposed to represent an accelerated aging phenotype in the arterial wall in which the biological age of arteries outpaces chronological age, and it may occur at higher rates in individuals with cardiometabolic diseases or in the setting of other age-related diseases^54^. Previous evidence has shown PWV can be used to predict arterial wall aging and has been correlated with increased vascular age as measures by the Framingham and SCORE 10-year risk of fatal CVD scores^55,56^; similarly, PWV has been shown to identify subjects with elevated vascular age and has been used as a cardiovascular marker to predict physiological aging rates in humans^57,58^. Further studies are needed to explore the role of PWV as a measure of biological age or as a component of aging biomarkers to characterize it as a relevant maker of accelerated vascular aging^59,60^.

### Arterial stiffness and arterial hypertension

AS is a pivotal pathophysiological precursor of SAH^4^. Research indicates that increased cf-PWV often precedes BP rises, and aortic stiffness assessed clinically is linked to faster BP increases and higher risk of incident SAH^8^. Notably, two major studies reveal that AS progresses independently of baseline BP levels, implying that stiffness itself may play a causative role in the development of sah^4,61,62^. Isolated systolic hypertension is the most common type of SAH in older adults, and is usually caused by increased aortic AS associated with aging^63^. Furthermore, AS is frequently present in individuals with SAH and has been associated with a higher risk of IR and diabetes^6^. In healthy arteries, the stiffness difference between central and peripheral arteries creates backward BP waves, therefore reducing the amount of energy that reaches small blood vessels, providing protection to the microcirculation^10^; however, this difference decreases in AS, leading to more pressure being transmitted to small peripheral arteries and to the microcirculation, increasing the risk of microvascular damage^64^. Both high PWV and an enlarged aortic diameter are important contributors of SAH and strong predictors of cardiovascular risk in younger and older adults^4,8^.

AS reduces diastolic BP due to the premature return of pressure waves to the heart during systole, which diminishes the driving force for coronary blood flow during relaxation^65^, and may hinder the effectiveness of pharmacological treatments aimed at improving coronary circulation. Furthermore, the accompanying rise in systolic BP increases LV workload and oxygen demand, which also impacts treatment outcomes^66^. AS also plays a critical role in increasing the risk of SAH-related complications by promoting damage to target organs such as the heart, kidneys, brain, and peripheral arteries^66,67^. Increased AS worsens cardiovascular prognosis, even in hypertensive patients receiving treatment. While BP-lowering therapy can reduce aortic stiffness in the short term through wall unloading, persistent AS indicates a poor prognosis despite treatment^68^.

### Arterial stiffness and dyslipidemia

The development and progression of AS is closely linked to lipid metabolism^69^. Standard blood lipids, non-traditional lipid markers, and lipid ratios are all associated with increased AS^70^. Elevated lipid levels stimulate the release of cytokines and adhesion molecules from leukocytes, which then enable these cells to attach to the vascular endothelium and penetrate the *intima*, contributing to chronic inflammation and thickening of the arterial wall. Furthermore, lipid accumulation induces oxidative and nitrosative stress, which accelerates arterial stiffening^71^. Arteriosclerosis, characterized by the thickening and hardening of arterial walls, largely results from structural and functional shifts, such as the replacement of elastic fibers with collagen, damage to muscle fibers, and calcium deposits^72^. This condition shares risk factors with atherosclerosis, including aging, oxidative and nitrosative stress, high BP, and inflammation^69^. Multiple studies have shown a significant association between AS as measured by ba-PWV and elevated triglycerides (TG) and total cholesterol levels^71,73^. While the relationship between PWV and low-density lipoprotein cholesterol (LDL-C) remains unclear, TG are consistently associated with AS and are often involved in early CVD, especially in individuals with low LDL-C levels^69^. On the other hand, lipoprotein(a) also correlates with PWV, however, its independent association with AIx suggests a more specific impact on the peripheral arterial tree^74^. When comparing lipid parameters, studies suggest that lipid ratios, such as the TG-to high-density lipoprotein cholesterol (HDL-C), are more strongly linked to high PWV than individual lipid values alone, particularly in those with high TG and low HDL-C levels^75,76^. Moreover, AS is important for the progression of atherosclerosis in familial hypercholesterolemia (FH), contributing to higher cardiovascular risk and earlier coronary stenosis. Although some studies show increased PWV and other stiffness measures in FH patients, their clinical significance remains unclear. Additional assessments of AS in individuals with FH and familial combined hyperlipidemia (FCHL) revealed important distinctions between the two groups; specifically, patients with FCHL exhibited higher AS compared with FH, which is also in accordance to a higher burden of cardiometabolic risk factors in FCHL compared to FH^77^. Larger studies are needed to clarify the link between AS, FH, FCHL and other primary dyslipidemias, particularly for improving early detection and guiding treatment approaches for the reduction of cardiovascular risk^78^.

### Arterial stiffness and alterations in glucose metabolism

CKMS and obesity are often linked to IR and hyperinsulinemia, which have been shown to increase the risk of AS in states of impaired glucose metabolism^6,79^. However, the relationship between AS and IR is complex and not yet fully understood^6,80,81^. The association of CKMS and elevated AS is also affected by age, which may raise the risk for both AS and T2D^82,83^. Elevated levels of inflammatory markers such as IL-6 and TNF-α are commonly found in obesity and CKMS, potentially contributing to IR, although the precise mechanisms are still unclear^84^. Increased pulse pressure during AS may lead to endothelial dysfunction, which can worsen IR^85^. AS damages capillaries, impacting organs with low resistance like the endocrine pancreas, affecting insulin and glucagon secretion^86^. Furthermore, microvascular changes in skeletal muscle and liver may impair glucose metabolism and insulin sensitivity, disrupting metabolic balance^6,34,63^.

Diabetes is recognized as a major risk factor for CVD, kidney damage, and organ dysfunction. Research has shown that PWV is increased in individuals living with prediabetes and T2D compared to individuals with normal fasting plasma blood glucose^61,87^. Similarly, individuals with abnormal glucose tolerance had stiffer arteries compared to those with normal glucose tolerance. Although the relationship between AS and diabetes might be bidirectional, its temporality remains unclear^6,87,88^. Individuals living with diabetes may be at a higher risk of developing AS with increasing HbA1c levels, and patients with both diabetes and AS face higher risk of all-cause mortality^61,89–91^; conversely, research has shown that individuals in the highest cf-PWV quartile have three-fold higher risk of developing diabetes compared to those in the first quartile^92^. A recent longitudinal study found that participants with SAH and AS were at a higher risk of developing T2D compared to those in other groups. Specifically, individuals with SAH but normal AS had the lowest diabetes risk, while those with elevated AS exhibited a substantially higher likelihood of developing T2D^74^.

HbA1c levels are associated with greater AS both in patients with and without T2D, suggesting a relevant role for chronic hyperglycemia and IR in promoting structural changes in the arterial wall^93,94^. Hyperglycemia, evaluated by fasting blood glucose, HbA1c, and 2 h blood glucose post-oral glucose tolerance test, independently contributes to the increase in central AS, as measured by cf-PWV^95^. The presence of multiple altered glycemic markers leads to an even greater rise in cf-PWV, suggesting a cumulative effect on arterial health^95^. Growing evidence further links increased AS to prediabetic states, such as impaired fasting glucose, glucose intolerance, and IR^96,97^. In fact, prediabetes is associated with endothelial dysfunction, a key factor in the development of AS^98^. This dysfunction is largely driven by hyperglycemia-induced oxidative stress, which reduces endothelial nitric oxide synthase expression and impairs NO availability and metabolism, contributing to vascular damage^99^. Studies have shown that even in the absence of overt diabetes, elevated fasting glucose and HbA1c levels are associated with greater AS^94^. However, this relationship is not entirely consistent, as some studies have failed to find a significant association between impaired fasting glucose and AS, indicating that further research in this area is needed.

### Arterial stiffness and adiposity

AS in individuals with obesity is a significant and independent risk factor for CVD, cognitive decline, and CKD^34^. In obesity, changes in adipose tissue metabolism cause the release of various bioactive molecules and hormones, including inflammatory cytokines TNF-α, IL-6, angiotensinogen, aldosterone, and leptin. Elevated levels of proinflammatory molecules decreases insulin sensitivity in blood vessels and draw immune and proinflammatory cells to the vascular system, which can lead to AS^3^. Other elements contributing to this condition are stiffening of endothelial and VSMC, alterations in ECM, inflammation of the adipose tissue surrounding blood vessels, and immune-cell dysfunction^34^. Recent evidence on arterial distensibility in individuals living with obesity have shown an association between obesity, particularly abdominal adipose tissue, and increased AS^100^. Adipose tissue distribution plays a key role in cardiovascular risk, with visceral adipose tissue being associated with a higher risk for CVD and AS compared to subcutaneous adiposity, due to higher levels of proinflammatory adipokines produced by visceral adipose tissue depots^101^. In states of IR, increased lipolysis in visceral adipose tissue results in increased free fatty acid release, which can interfere with insulin’s ability to promote glucose uptake and support metabolic signaling in both adipose and vascular tissues^102^. This disruption in insulin signaling also lowers NO production, which contributes to endothelial dysfunction and hardening of endothelial and VSMCs^34^. Premenopausal women typically benefit from the cardiovascular protective effect of estrogens, but this advantage diminishes in the context of obesity, IR, or T2D, and is closely associated with increased AS^34^. Impaired insulin sensitivity in adipose tissue significantly promotes arterial stiffening by activating RAAS and increasing angiotensin II levels, which contribute to vascular remodeling and AS. This effect is particularly strong in diet-induced obesity, where activation of mineralocorticoid receptors in vascular cells plays a key role in AS, especially in females^102,103^.

Adiponectin and leptin, two key adipokines produced by white adipose tissue, have opposing effects on AS^104,105^. Adiponectin helps reduce AS by enhancing NO availability, which regulates vascular tone, and by inhibiting the proliferation and migration of VSMC, thus preventing excessive vascular remodeling^106^. However, in conditions like obesity, adiponectin levels are often low, hindering endothelial function and contributing to vascular damage and AS, particularly in the presence of low-grade inflammation and arterial hypertension^107^. In contrast, elevated leptin levels, commonly seen in obesity and SAH, promote VSMC proliferation, endothelial oxidative stress, and reduced NO availability, all of which impair aortic function. Leptin also activates RAAS, increasing BP and worsening endothelial dysfunction, furthering AS^105^. Therefore, while adiponectin exerts protective effects on vascular health, leptin exacerbates AS, emphasizing the complex role of adipokines in AS progression^108^.

## Evidence of cardiometabolic risk factor management impact on arterial stiffness

**Fig. 3:**
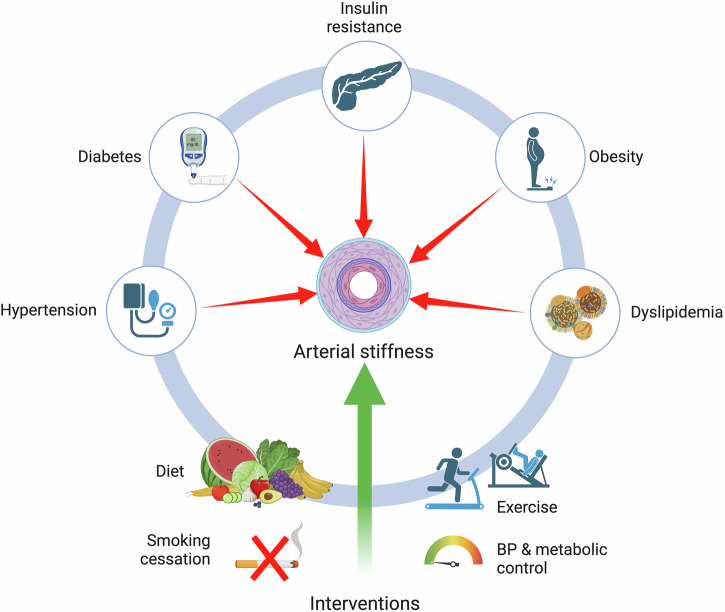
Contribution of cardio-metabolic diseases to the risk and progression of AS (red arrows), as well as the potential lifestyle changes and health interventions which have shown potential benefits in ameliorating AS in patients living with cardio-metabolic diseases (green arrow). Created in BioRender. Bello Chavolla, O. (2025) https://BioRender.com/r74b925.

As mentioned in earlier sections, AS is recognized as a marker of cardiovascular risk in cardiometabolic conditions, particularly among high-CVD risk groups^4^. Factors such as obesity, dyslipidemia, inflammation, oxidative stress, and IR are commonly associated with SAH and T2D, and with increased risk of micro and macrovascular complications^34,61^. Several studies have shown a link between increased AS and inflammation, and research suggests these changes may be reversible if the inflammatory condition is treated. Cardiometabolic risk factors can also accelerate AS progression, but if kept within low, non-disease levels, they may lead to a slower progression rates^41^. Early detection of vascular stiffening, along with its management through medications and lifestyle modifications, could help minimize future complications. Preventive drugs, including lipid-lowering and antidiabetic medications, have also been shown to impact AS independently of BP^4,9^. Lifestyle modifications and health interventions such as smoking cessation^109^, dietary changes, weight loss, physical activity, and adequate metabolic and BP control have been shown to positively impact AS, which highlights the importance of these interventions to manage cardiometabolic risk factors and AS^41,110^ (Fig. 3).

### Impact of lifestyle changes on arterial stiffness

#### Dietary changes

Lifestyle interventions play a fundamental role in AS management. Weight loss regimens based on diet and lifestyle changes have demonstrated benefits on vascular health, and losing an average of 8% of initial body weight through dietary adjustments has been shown to improve PVW^79^. Some of the most important strategies in dietary interventions which influence AS include caloric restriction (CR), reducing ingestion of saturated fats, simple carbohydrates, and sodium, and incorporating vitamins or antioxidative nutrients like polyphenols^111^. CR can improve age-related conditions and has demonstrated beneficial effects across various physiological and pathological processes which may help delaying declines in heart function, reducing fibrosis, risk of CVD, and AS^79^. Research has demonstrated a connection between CR, reduced body weight and improvement in AS^112^. Reducing carbohydrate intake induces favorable metabolic changes, contributing to decreases in AS, as shown by a reduction in PWV in women^113^. Both DASH and Mediterranean diets have been shown to positively impact vascular health^114,115^. The DASH diet is characterized by a high intake of vegetables, fruits, low-fat dairy, and lower sodium intake, which can positively impact AS^116,117^. A meta-analysis across various populations showed that an average reduction in sodium intake of 89.3 mmol/day (or 5.2 grams of salt per day) is linked to a 2.84% decrease in PWV^118^. The Mediterranean diet prioritizes vegetables, seeds, cereals, legumes, whole grains (brown rice and oats), lean meats, fish, and olive oil, and a 12-week daily regimen of omega-3 fatty acid supplementation has been shown to reduce PWV in older, but not in healthy young adults^119^.

#### Physical activity and arterial stiffness

The benefits of physical activity on AS have been well documented^47^; however, the impact of resistance training on BP and AS can vary greatly, depending on exercise intensity, chronological age, and overall health levels of an individual^110^. A systematic review and meta-analysis of randomized controlled trials found that aerobic exercise training resulted in a more prominent decrease in ba-PWV (β = −1.01 m/s) compared to cf-PWV (β = −0.39 m/s)^120^; furthermore, low-to-moderate intensity resistance training has shown to produce sustained reductions in PWV (β = −0.39 m/s)^121^, and has been beneficial for AS in patients with kidney disease or established CVD, as well as in normotensive and hypertensive individuals^120–122^. The effect of resistance training on PWV also varies based on the intensity of the exercise. High-intensity resistance training did not significantly impact PWV; in contrast, low-to-moderate intensity training led to significant improvements in PWV (β = −0.34 m/s). In young men and women, combined exercise enhances the benefits of physical activity on AS and reduces carotid artery stiffness, likely due in part to reductions in SBP^123^. Mechanisms involved in the observed vascular improvements of physical activity likely work synergistically^123^. Increased bioavailability of NO and reduced inflammation may drive a significant decrease in AS after combined exercise. Because AS increases with chronological age, regular aerobic activity can counter this progression by reducing BP^110^. This reduction in BP eases mechanical stress, balances metabolic and neurohormonal factors, and decreases inflammation, helping arteries retain their elasticity. In some cases, regular aerobic exercise has been shown to reverse existing stiffness, highlighting its powerful impact on vascular health^47^.

Cardiorespiratory fitness (CRF) is a relevant marker for cardiometabolic health which is inversely and independently associated with risk of all-cause mortality and CVD outcomes^124–127^. Notably, the impact of physical activity on CRF is one of the most relevant predictors of its cardiovascular benefits^125,126^. Higher cf-PWV and ba-PWV values have been associated with decreased CRF, particularly in patients living with metabolic syndrome, obesity, SAH or T2D^128–130^, indicating that AS may lead to lower CRF in patients living with cardiometabolic diseases. Interestingly, aerobic exercise consistently increases CRF and vascular health by increasing aerobic capacity and reducing BP and AS, and in patients with cardiometabolic diseases and individual CKMS components, combined aerobic and resistance training improve CRF in addition to its metabolic benefits^131–133^. A potential obstacle to implementing physical activity as a treatment for AS is the association between high AS and reduced exercise capacity, as it may occur in the setting of cardiometabolic diseases^134,135^. This limitation arises from two hemodynamic effects; first, AS raises systolic BP, which increases the heart’s workload and its oxygen requirements, and second, it reduces the Windkessel effect in large arteries, leading to less blood being stored in these arteries during diastole and decreasing end-organ perfusion^47,110^. In the setting of increased oxygen demand during physical activity, these observed hemodynamic changes during AS may decrease exercise capacity and thus limit its utility as a clinical intervention^134^. Additional research is required to investigate optimal physical activity regimens in patients with reduced CRF in the setting of AS, and its impact on reducing AS in patients with comorbid cardiometabolic diseases.

### Impact of cardiometabolic disease management on arterial stiffness

AS accelerates the onset and progression of CVD and serves as a marker of increased CVD risk, particularly in individuals with CKMS, obesity, IR, or diabetes^6^. In adults, the presence of a high number of CKMS components correlates with elevated ba-PWV and progression of AS^41^; in fact, evidence suggests that AS may precede the onset of individual CKMS components^8,136^. Weight loss, reduced salt intake, and exercise are strategies that not only help control BP and cardiometabolic diseases but have also been investigated for their potential to improve AS. Part of the effectiveness of these interventions is linked to improvements in cardiometabolic risk factors^68^.

#### Impact of blood pressure management on arterial stiffness

Recent anti-hypertensive drugs offer benefits beyond just lowering BP, and reducing AS with these drugs is expected to provide additional advantages apart from BP reduction^4^. Evidence from a longitudinal analysis of the Kailuan study showed that antihypertensive medications may have a primary influence in PWV, even before any benefits are observed in BP levels^13^. Furthermore, ba-PWV prior to treatment initiation in adults with SAH has been shown to influence the BP lowering effects of antihypertensive medications, whereby higher AS was usually indicative of decreased BP response to short-and long-term treatment^137^. PWV is an important indicator of organ or tissue-specific damage, making it an invaluable tool for identifying arterial injury^11^. AS measurement can provide a strong rationale for initiating antihypertensive therapy; furthermore, the potential bidirectional causal relationship between SAH and AS highlights the importance of considering the presence of AS as a criterion when deciding to initiate antihypertensive treatment^4,66^. Notably, uncontrolled systolic and diastolic BP levels in adults with SAH is associated with higher ba-PWV, indicating that AS may be a clinically useful marker for uncontrolled SAH^138^. The Systolic Blood Pressure Intervention Trial (SPRINT) showed improved survival rates in hypertensive patients whose PWV decreased after treatment, independently of any reduction in SBP^139^. These results collectively emphasize the role of PWV as a crucial parameter in tailoring antihypertensive strategies to optimize cardiovascular outcomes^4^.

Specific antihypertensive drug classes may have differential impacts on PWV and AS. For example, angiotensin-converting enzyme inhibitors (ACEI) have been shown to be more effective in reducing PWV compared to placebo, angiotensin-receptor blockers (ARB), calcium-channel blockers (CCB), β-blockers, diuretics, and a combination of ACEI and ARB, with no significant influence of BP changes on their effect on PWV^140^. Evidence on the impact of ARB on AS suggests similar effects to other antihypertensive agents, largely attributed to their ability to relax VSMCs within primary major elastic arteries, and improve the AIx^141,142^. The use of CCB and β-blockers have shown heterogeneous effects on AS; while their impact have been beneficial compared to placebo, these antihypertensive drug classes appear to be less effective at reducing PWV compared to ACEI and ARB^143,144^. The effectiveness of RAAS inhibition on AS management may make ACEI and ARB better alternatives compared to other antihypertensive drug classes in the management of AS.

#### Impact of weight loss and obesity management on arterial stiffness

Weight loss achieved through dietary and lifestyle changes has a direct impact on reducing AS. For instance, a four-month reduction in body mass significantly improves aortic and carotid stiffness in subjects living with overweight and obesity^79,84,108^. Even small reductions in weight can enhance glycemic control, lower BP and triglyceride levels, and increase HDL cholesterol, underscoring the importance of weight management in vascular health^6^. Weight loss with average body weight reductions of 8% may lead to a PWV reduction estimated at −0.32 m/s, with more pronounced (but not statistically significant) reductions observed in ba-PWV compared to cf-PWV (−0.48 m/s vs. −0.35 m/s). Modest reductions ranging 5% to 10% of body weights can lead to substantial improvements in AS particularly through its effect in BP reductions^145^. Therefore, AS may be managed through the impact of lifestyle interventions on weight loss, particularly in individuals with obesity.

The impact of bariatric surgery on AS in individuals with obesity has also been documented, showing declines of up to −0.65 m/s on PWV post-surgery, independent of baseline BMI and the duration of follow-up^146^. Notably, the effects of bariatric surgery on AS may be in part due to their impact on other cardiometabolic risk factors, as the positive effect of bariatric surgery on PWV was not observed in metabolically-healthy adults with obesity in a recent study^147^. Interestingly, individuals with median increases of 104% on GLP-1 levels post-bariatric surgery compared to baseline showed greater decreases in cf-PWV compared to non-responders, indicating that changes in GLP-1 levels after bariatric surgery may be the best predictor of decreases in AS^148^. This evidence supports a potential role for pharmacological agents, including GLP-1 receptor agonists (GLP-1RA) on improving AS in individuals with obesity, particularly with the strong benefits of GLP-1RA on cardiovascular outcomes^149^; however, to date no study has shown conclusive evidence of their impact on AS or PWV in adults with obesity but without T2D^150^.

#### Management of diabetes and arterial stiffness

In T2D, persistent hyperglycemia and IR accelerate AS by damaging the endothelial glycocalyx and increasing inflammatory activity. This stiffness impairs vascular elasticity, raises PWV, and contributes to hypertension and left ventricular strain^65^. Management of T2D plays a key role in mitigating AS by reducing advanced glycation end-products and their interaction with their receptors^2^. When assessed together with glycemic control, individuals with good glycemic control still had a high risk of macrovascular complications when affected by moderate-to-severe AS; conversely, those with poor glycemic control had the highest risk with concomitant severe AS^151^. Evidence suggests that adequate glycemic control in patients with T2D also reduces PWV and attenuates the impact of AS on T2D^152,153^.

The role of specific medications for T2D in managing AS has also been investigated. Metformin has shown modest benefits in improving the AIx and reducing aortic PWV, ba-PWV and BP primarily through its effect on peripheral insulin sensitivity, increased adiponectin levels, and weight loss^154,155^. Pioglitazone and rosiglitazone have also shown improvements in endothelial function and AS by increasing arterial flexibility and causing a secondary amplification of adiponectin levels, and may be superior for improving endothelial function compared to placebo^156–158^. In a recent network meta-analysis of randomized controlled trials, thiazolidinediones, GLP-1RA and SGLT-2 inhibitors were shown to reduce PWV by up to −0.5 m/s compared to placebo, without significant benefits for sulfonylureas, metformin, DPP-4 inhibitors and insulin^158^. GLP-1RA and SGLT-2 inhibitor therapies can improve the glycocalyx integrity by reducing inflammation and thus enhancing arterial compliance by targeting both metabolic dysfunction and vascular health providing protection against cardiovascular complications^65^. These drugs offer beneficial effects on BP, body weight, HbA1c levels, and lipid profiles. In a previous study, Ikonomidis et al. showed that combining SGLT-2 inhibitors and GLP-1RA for a 12-month treatment improved glycemic control in individuals with T2D and a steeper reduction in PWV in comparison to insulin and GLP-1RA alone, along with significant reductions in body weight and fat mass^159^. Despite recent evidence showing a potential benefit of SGLT-2 inhibitors and GLP-1RA on improving AS in patients with T2D, additional studies are required to evaluate whether this effect is conditional on glycemic control or potentially influenced by weight loss^160,161^.

#### Management of dyslipidemia and arterial stiffness

Statins confer beneficial properties for vascular function, such as anti-inflammatory and antioxidant effects which may contribute to managing AS^5^. A meta-analysis of randomized control trials showed that the use of simvastatin, rosuvastatin, lovastatin, fluvastatin, and atorvastatin was associated with slower aortic PWV^162^. Similarly, high-intensity statin therapies were shown to decrease PWV by −1.17 m/s, compared to −0.80 m/s with moderate-intensity and a non-significant −0.5 m/s for low-intensity statins regimes^163^. Interestingly, a network meta-analysis which compared the use of statins compared to physical activity for reducing AS showed higher surface under the cumulative ranking curve (SUCRA) for high- and moderate-intensity statin therapies (74% and 67%), but lower SUCRA for low-intensity regimes compared to high-intensity exercise (60% vs. 67%), indicating that physical activity may be considered as an alternative to low-intensity statins for managing AS^163^. Furthermore, a systematic review and meta-analysis explored the impact of adding PCSK-9 inhibitors to lipid-lowering interventions on arterial stiffness, identifying a reduction of −2.61 m/s in PWV after adding PCSK9 inhibitors to standard statin therapy. Interestingly, the impact of PCSK-9 inhibitor addition on AS showed a greater impact in men and a dependent effect for patients with higher baseline PWV values^164^.

## Conclusions and perspectives

AS is a risk factor and a relevant marker to evaluate CVD risk. Given the substantial overlap between the pathophysiology of AS, aging, and cardiometabolic diseases, follow-up and management of cardiometabolic diseases must consider their impact on PWV and AS to adequately manage cardiovascular risk in these populations. Individuals living with obesity, particularly abdominal obesity, IR, prediabetes, T2D, SAH, and primary and secondary dyslipidemias must be considered at higher risk of AS and should be evaluated using non-invasive techniques, particularly those without prior history of CVD given the evidence of their utility as a marker of vascular health^42^. Adequate metabolic and BP control may offer beneficial effects on AS for patients living with cardiometabolic disease; however, it is currently unclear whether serial measurements of PWV may be clinically useful, or whether reductions in PWV after therapeutic interventions may offer benefits relevant to micro or macrovascular outcomes in these patients^18,165,166^. Further randomized studies are required to evaluate the impact of specific interventions aimed at improving metabolic control on AS and, specifically, on whether these benefits translate to meaningful reductions on the risk of CVD outcomes.

Despite substantial evidence on the impact of lifestyle interventions on AS and PWV, further research is needed to understand the impact of managing specific macro and micronutrients on AS progression, as well as evidence from randomized controlled trials evaluating the impact of dietary interventions on AS and its potential modifiers including but not limited to Mediterranean and DASH diets, CR and intermittent fasting, which has not previously been explored in depth despite some planned trials primarily focused on time restricted eating^167^. The evidence of physical activity on AS is strong and it may confer stronger benefits for reducing PWV compared to some therapeutic interventions. However, whether the effect of specific physical activity prescription on cardiometabolic diseases may offer additional benefits requires additional studies. Many of the potential benefits of diet and physical activity prescription on AS could be attributed to reductions of between 5–10% of body weight, which may also confer additional benefits in improving other metabolic parameters. Additional research is needed to investigate whether the use of GLP-1RA may be useful to reduce AS in adults living with obesity but without type 2 diabetes.

Specific treatment of cardiometabolic diseases has also shown benefits in managing AS. In patients living with SAH, adequate BP control may require additional management of AS. Evidence suggests that the use of ACEIs and ARBs may provide benefits in reducing PWV and AS even before benefits in BP are observed, and which may be superior to other antihypertensive medications. Monitoring PWV may be useful to assess cardiovascular risk as part of the management of SAH and may lead to greater benefits in cardiovascular outcomes compared to BP monitoring alone; however, this will need to be confirmed in randomized controlled studies. In the case of T2D, the use of SLGT-2 inhibitors and GLP-1RA may be useful to achieve glycemic control, weight loss and reductions in AS and PWV; this may be useful given that AS has been associated with worse outcomes in patients with diabetes independent of glycemic control. Whether management of AS in patients with T2D offers additional benefits for micro and macrovascular complications requires further evidence from prospective observational studies and randomized controlled trials. Finally, moderate and high intensity statin therapy has shown substantial benefits in improving AS and reducing PWV in patients with dyslipidemia and addition of PCSK-9 inhibitors may provide benefit, especially for patients with elevated PWV as it occurs in those living with primary dyslipidemias.

Given the relevance of AS as mechanisms of vascular aging, its influence on cardiovascular health and its utility as a prognostic marker for CVD, PWV should be considered as a potentially useful non-invasive measure of vascular health in patients living with cardiometabolic diseases. Substantial evidence supports the clinical relevance of PWV measurement in investigating CVD, monitoring SAH, assessing the effectiveness of cardiometabolic risk factor management, and even evaluating cerebral blood flow to estimate stroke and dementia risk^60^. However, its routine use in clinical settings remains limited due to technical and scientific challenges^60,168,169^. Further research is needed to validate the accuracy and consistency of non-invasive PWV measurements compared to invasive methods and to establish their predictive value for relevant outcomes independent of age and BP levels. Additionally, studies should explore the ability of PWV to detect both overt and subclinical vascular changes, which are may be useful for cardiovascular risk stratification, therapy initiation, and monitoring^60,169^. As research in this field rapidly evolves, future studies should prioritize assessing the clinical utility of PWV measuring and monitoring, as well as the therapeutic and prognostic implications of AS management in patients with cardiometabolic diseases. Clarifying the role of AS in the pathophysiology of CKMS and its implications for tracking and managing individual cardiometabolic risk factors will further enhance its integration into clinical practice.

## Data Availability

No datasets were generated or analysed during the current study.

## List of abbreviations

**ACEI**: Angiotensin-Converting Enzyme Inhibitors
**AIx**: Augmentation Index
**ARB**: Angiotensin-Receptor Blockers
**AS**: Arterial Stiffness
**ba-PWV**: Brachial-Ankle Pulse Wave Velocity
**BP**: Blood Pressure
**CAVI**: Cardio-Ankle Vascular Index
**CCB**: Calcium-Channel Blockers
**cf-PWV**: Carotid-Femoral Pulse Wave Velocity
**CKMS**: Cardiovascular-Kidney Metabolic Syndrome
**CKD**: Chronic Kidney Disease
**CRF**: Cardiorespiratory fitness
**CVD**: Cardiovascular Disease
**DASH**: Dietary Approaches to Stop Hypertension
**DPP-4 inhibitors**: Dipeptidyl Peptidase-4 Inhibitors
**ECM**: Extracellular Matrix
**FCHL**: Familial Combined Hyperlipidemia
**FH**: Familial Hypercholesterolemia
**GLP-1**: Glucagon-Like Peptide-1
**GLP-1RA**: GLP-1 Receptor Agonists
**IR**: Insulin Resistance
**LV**: Left Ventricular
**MCP-1**: Monocyte Chemoattractant Protein-1
**MFG-E8**: Milk Fat Globule-Epidermal Growth Factor 8
**MRI**: Magnetic Resonance Imaging
**NADPH**: Nicotinamide Adenine Dinucleotide Phosphate
**NO**: Nitric Oxide
**PCSK-9 inhibitors**: Proprotein Convertase Subtilisin/Kexin Type 9 Inhibitors
**PPP1CB**: Protein Phosphatase 1 Catalytic Subunit Beta
**PRKA**: Protein Kinase A
**PWV**: Pulse Wave Velocity
**RAAS**: Renin-Angiotensin-Aldosterone System
**ROS**: Reactive Oxygen Species
**SAH**: Systemic Arterial Hypertension
**SGLT2 inhibitors**: Sodium-Glucose Cotransporter 2 Inhibitors
**SUCRA**: Surface Under the Cumulative Ranking Curve
**T2D**: Type 2 Diabetes Mellitus
**TGF-β**: Transforming Growth Factor Beta
**TNF-α**: Tumor Necrosis Factor Alpha
**VSMC**: Vascular Smooth Muscle Cells
